# Interferon-*γ* derived from cytotoxic lymphocytes directly enhances their motility and cytotoxicity

**DOI:** 10.1038/cddis.2017.67

**Published:** 2017-06-01

**Authors:** Purnima Bhat, Graham Leggatt, Nigel Waterhouse, Ian H Frazer

**Affiliations:** 1 The University of Queensland Diamantina Institute, Translational Research Institute, 37 Kent Street, Woolloongabba, QLD 4102, Australia; 2 Medical School, John Curtin School of Medical Research, Garran Rd, Australian National University, Canberra, ACT 2601, Australia; 3 QIMR Berghofer Medical Research Institute, 300 Herston Road, Brisbane, 4006, QLD. Australia

## Abstract

Interferon gamma (IFN*γ*) is a key moderator of cell-mediated immunity with diverse, mainly pro-inflammatory actions on immunocytes and target tissue. Recent studies have shown it may enhance anti-tumor and antiviral effects of CD8 T cells. Here we investigate the mechanisms by which IFN*γ* mediates CD8 T-cell cytotoxic function. We show that *in vivo*, antigen-specific CD8 T cells that produce INF*γ* are necessary to effect rejection of skin grafts expressing OVA as a transgene in keratinocytes. The ability of CD8 T cells to produce IFN*γ* enhanced their ability to migrate to the site of antigen-presenting skin cells. By *in vivo* imaging, we show that CTL motility, particularly speed, during graft rejection was enhanced by locally available IFN*γ*. We then used a reductionist two-cell model of CTL effectors and keratinocyte targets to investigate the effects of locally available (paracrine) and CTL-producing (autocrine) IFN*γ* on the motility behavior and killing ability of the CTL. Using live-cell imaging by prolonged time-lapse microscopy of primary effector CD8 T cells and antigen-expressing primary keratinocyte targets, we show that CD8 T-cell cytotoxic function and motility is enhanced by locally available IFN*γ*. Conversely, deprivation of either autocrine or paracrine IFN*γ*, or blockade of IFN*γ* signaling to CTL markedly reduced their cytotoxic function, their kinematics, and effector cell survival. We conclude that *in vitro* and *in vivo,* autocrine production of IFN*γ* by CTL enhances their motility and promotes killing of primary target keratinocytes. The absolute need for local IFN*γ* to enable cytotoxic CD8 T-cell function is of significance for immunotherapy for chronic viral infection and for cancer.

Cytotoxic CD8 T lymphocytes (CTL) are found in many solid tumors and provide an attractive target for immunotherapeutic manipulation.^1, 2^ However, despite their presence, they appear to function sub-optimally in effecting target cell lysis. Inhibiting CTL regulatory mechanisms have shown promise as potential adjuvant cancer therapies. Vaccination together with TGF-*β* blockade,^3^ IFN-*α* therapy^4^ or inhibition of CTLA-4,^5^ or of PD-1/PD-L1 interactions,^6^ have enhanced effector T-cell function in melanoma. Local cytokines such as IL-12 have been shown to promote intra-tumoural CD8 T-cell function.^7, 8^ A favorable ratio of effector T cells to regulatory T cells is associated with a better prognosis, suggesting that CTL may play a role in controlling many malignancies. Human trials of immunotherapy in which there is marked activation of local effector T-cell function and inhibition of local regulatory T cells^9^ have shown benefit.

IFN*γ* is released in large amounts by macrophages, activated CD8 T cells, natural killer T cells, and Th1 CD4 T cells. Its actions are varied, and tissue dependent; the IFN*γ* receptor (IFN*γ*R) heterodimer is widely expressed on immune and non-immune cells.^10, 11^ Early in inflammation, it is a potent chemoattractant released by activated tissue macrophages, promoting recruitment of neutrophils, and T cells through paracrine signaling. It markedly increases T-cell-mediated killing by upregulating MHC-I expression on target cells, and may promote target cell differentiation and death directly.^12, 13^ IFN*γ* skews the helper T-cell response towards a Th1 profile, but may be inhibitory in some infection models by suppressing IL-17 and reducing neutrophil chemotaxis.^14, 15, 16^ Studies enhancing the expression of IFN*γ* by CD8 T cells have shown improved anti-tumor responses *in vivo* in several mouse models.^17, 18^

IFN*γ* affects a variety of intracellular events in CD8 T cells via the IFN*γ*R. It induces IL-12R expression^19^ and in murine models of infection, CTL proliferation and immunodominance appear to rely on IFN*γ*.^20, 21^ IFN*γ* may enhance the ability of CTL to kill via Fas/FasL in the absence of perforin.^22^ However, it may also directly increase T-cell apoptosis, and reduce proliferation.^23^ Thus reports on the actions of IFN*γ* on CD8 T cells vary. In skin, IFN*γ* appears to be essential to promoting T-cell migration to sites of inflammation, even in sterile conditions.^24, 25^ We have shown IFN*γ* to be essential in mediating rejection of skin grafts expressing ovalbumin,^26^ but it is suppressive of CD8 T-cell function when other antigens are expressed.^27^

We have previously shown that the cytotoxic ability of CD8 T cells was associated with their kinematics in target tissue.^28^ Here we examine the mechanisms by which local IFN*γ* affects CD8 T-cell motility and modulates the ability of CD8 effector T cells to kill keratinocytes (KC) expressing non-self antigen. *In vivo*, CTL needed to produce IFN*γ* to achieve skin graft rejection and IFN*γ* promoted CTL motility in tissue. *In vitro,* signaling by IFN*γ* increased CD8 T-cell motility and speed, and markedly increased antigen-specific contact-mediated T-cell killing. We show IFN*γ* enhances the cytolytic ability and the kinematics of CTL both by paracrine and autocrine mechanisms of signaling.

## Results

### IFN*γ*-producing CD8 T cells are required for graft rejection

**Figure 1:**
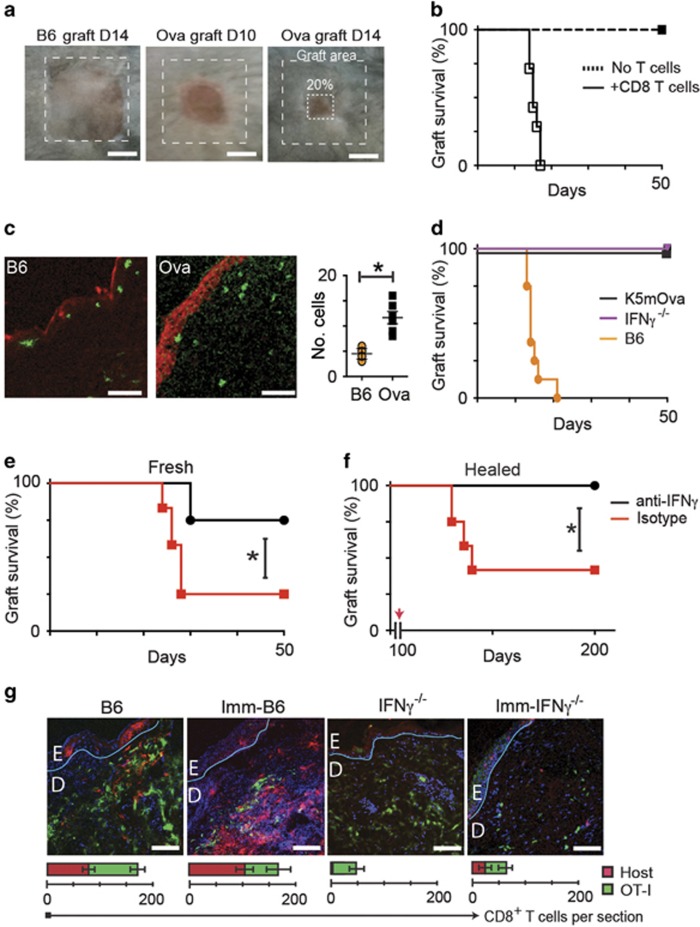
Interferon-*γ* is required for skin graft rejection. Ear skin from B6 or K5mOVA donor mice was grafted on the flanks of B6 recipients. (**a**) 80% graft loss was denoted as rejection. (**b**) OVA skin grafted onto Rag1^−/−^ mice with or without transferred 10^6^ naive CD8 T cells. (**c**) Section of OVA grafts onto B6 or OVA mice at day 10 stained for caspase-3 (red), CD8 (green; Bar, 100 *μ*m); quantified per field of view. Data pooled from 2 experiments, error bars are SEM. (**d**) OVA transgenic grafts were placed on K5mOva, IFN*γ*^−/−^ or B6 recipients. Graph shows graft survival. (**e**) IFN*γ*^−/−^ recipients received pre-primed CD8 cells by adoptive transfer, and were treated with anti-IFN*γ* or isotype antibody 48 h prior to grafting of OVA skin, and weekly thereafter. Graph shows graft survival (*P<0.05, Mantel–Cox test). (**f**) OVA skin grafts were placed on IFN*γ*^−/−^ mice for 100 days. Recipients were then adoptively transferred with IFN*γ*-competent CTL, and anti-IFN*γ* or isotype antibody as in (**e**). (*P<0.001, Mantel–Cox test). (**g**) EGFP^+^ OT-1 CD8 T cells were adoptively transferred into OVA-naive B6 mice, OVA-immunized B6 mice, OVA-naive IFN*γ*^−/−^ mice, or into OVA-immunized IFN*γ*^−/−^ mice, before placing OVA skin grafts. Grafts were harvested after 10 days and stained for CD8. Confocal images were taken at graft edges and have been pseudocoloured to show native CD8 cells (red), transferred cells (green) and nuclei (blue). The basement membranes have been drawn in teal. (Bar, 100 *μ*m). The average total cell numbers of T cells in each field from duplicate fields of two samples from a typical experiment is indicated in the graphs below. Error bars are S.D.

Rejection of antigen-expressing skin grafts is a model of CTL-dependent immunopathology. To examine the role of IFN*γ* in effector function of T cells against epithelial cells *in vivo*, we grafted skin expressing OVA onto B6 mice. These grafts are rejected at around 14 days (Figures 1a and b). At this time, the grafts can be seen to contain large numbers of infiltrating CD8 T cells, and extensive expression of caspases in keratinocytes (Figure 1c). By contrast, non-rejecting isografts showed minimal cellular infiltration and minimal expression of activated caspases, confirming that infiltrating antigen-specific CD8 T cells play a role in mediating the caspase-dependent death of keratinocytes that leads to graft failure.

OVA skin grafts placed on IFNγ^−/−^ recipient mice were not rejected (Figure 1d). This might reflect failure of T-cell activation, as IFN*γ* facilitates *in vivo* priming of naive T cells, or a requirement for IFN*γ* to enable T-cell function. We transferred 10^6^ OVA-primed CD8^+^CD44^high^ CD8 T cells to IFN*γ*^−/−^ mice bearing OVA skin grafts, either placed at the time of T-cell transfer or healed-in for 100 days. Recent OVA grafts were promptly rejected in animals with transferred primed T cells, but not in animals comprising only cells naive to OVA (Figures 1e and f) suggesting IFN*γ*-deficiency compromises T-cell activation. Healed-in OVA grafts showed inflammation and graft shrinkage after transfer of primed T cells, with 7 of 12 rejected (Figure 1f). Administration of anti-IFN*γ* antibody negated the effects of the transferred cells.

We tested whether IFN*γ* was required to recruit pre-primed T cells to effect rejection. We transferred 10^6^ EGFP^+^CD8^+^CD44^high^ OT-1 effector T cells from mice primed by immunization with OVA into OVA-naive mice that were either IFN*γ*-competent or IFN*γ*^−/−^, thus providing in each animal a primed and an unprimed population of T cells. Also, these effector T cells were transferred into mice that had been immunized with OVA thus providing two primed populations of T cells: host and transferred. These mice recipient of T cells were then grafted with OVA skin. Grafts on unimmunized IFN*γ*^−/−^ animals recruited fewer cells than grafts on immunized IFN*γ*-competent animals. Immunization of IFN*γ*^−/−^ mice with OVA was effective in facilitating recruitment of host cells to OVA grafts (Figure 1g). These data confirm that host IFN*γ* facilitates effective trafficking of antigen-specific CD8 T cells and may contribute to CTL activation.

### T-cell motility in tissue increases with rejection

**Figure 2:**
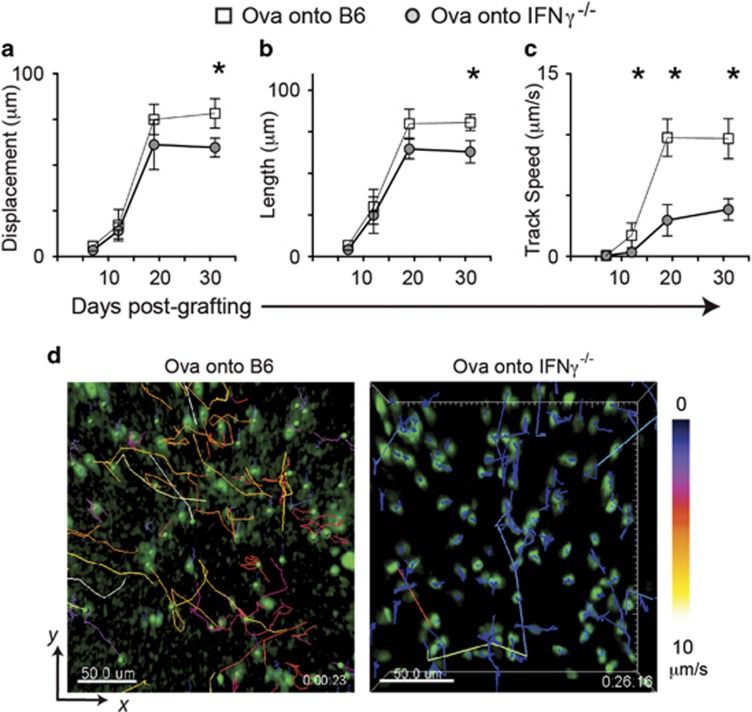
Motility of CTL in tissues is enhanced by IFN*γ*. EGFP^+^CTL were adoptively transferred into B6 or IFN*γ*^−/−^ mice before OVA skin was grafted. Post grafting, mice were anaesthetized and the graft edges imaged by 2-photon microscopy. Multiple sites per graft were imaged for 20-30 min each. EGFP^+^ cells were identified and tracked by software, and analyzed for CTL displacement (**a**), length of travel (**b**) and speed of travel (**c**) during the imaged time. Data are pooled from 4 regions imaged per graft, from 3 mice per time point. Averages are shown, error bars are SD. (*P<0.05) (**d**) Representative images of grafts on B6 recipients and IFN*γ*^−/−^ recipients at day 19 post graft placement. CTL have been identified and tracked with track color coding for average speed of cell movement over the period of imaging. Bar, 50 *μ*m (see also Supplementary Movies S1 and 2)

We have previously observed altered CD8 T-cell kinematics associated with acquisition of CD8 T-cell effector functions *in vitro*.^28^ We observed *in vivo* the motility of CTL in OVA grafts placed on IFN*γ*-deficient and IFN*γ*-competent mice. OVA grafts were placed on B6 or IFN*γ*^−/−^ mice recipient of EGFP^+^CD8^+^CD44^high^ OT-1 effector T cells. Grafts were examined by intravital imaging from day 7, after bandage removal. Early post-grafting, CTL in both B6 and IFN*γ*^−/−^ recipients displayed similar characteristics of immobility and arrest. By 12 days, when signs of graft rejection were visible, some CTL were active, showing increase in track displacement and length similarly in both B6 and IFN*γ*^−/−^ mice, but reduced speed in the absence of IFN*γ* (*P*=0.02; Figures 2a–c). OVA grafts on all B6 mice had completely rejected by 14 days. By day 19, there were marked differences between the kinematics of CTL in IFN*γ*-competent mice compared with their IFN*γ*-deficient counterparts, with the latter showing less speed (*P*=0.01), and trending to greater displacement (*P*=0.06) and track length (*P*=0.07; Figures 2c and d). These effects were even greater at 31 days after healing was complete (displacement *P*=0.02; length *P*=0.01; speed *P*=0.002). These data suggest that in the absence of acute inflammation, and after loss of antigen, locally available IFN*γ* enhanced kinematics of CTL *in vivo*.

### IFN*γ* enhances target killing

**Figure 3:**
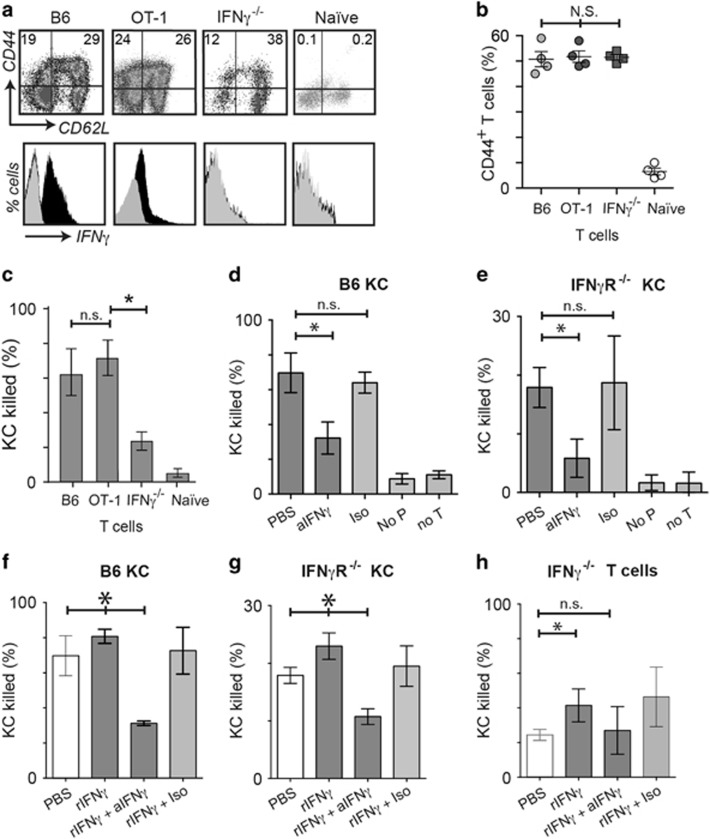
Exogenous IFN*γ* promotes KC killing by T cells via IFN*γ* receptor on target cells. (**a**) Flow cytometry analysis of splenic T cells gated by size, CD8 expression, and EGFP expression, from OVA-immunized OT-I, B6, IFN*γ*^−/−^, or OVA-naive B6 mice (black, anti-IFN*γ*; gray, isotype antibody). *N*=4. (N.S., not significant between groups, ANOVA.) (**b**) Percentage of total CD8 T cells from (**a**) that express CD44. *N*=4, error bars are SD. (**c**–**h**) These cells were co-cultured with SIINFEKL-loaded target keratinocytes derived from B6 (IFN*γ*R^+/+^) or IFN*γ*R^−/−^ mice in the presence of indicator dye for intracellular activated caspases, and imaged by time-lapse fluorescence microscopy. Target cell death was determined by analyzing movies using software to identify spots based on size (>12 *μ*m) and red fluorescence, and expressed as a percentage of the total number of KC at 30 h. Duplicate wells were averaged, *N*=4. Error bars are standard deviations. (*P<0.05, ‘NS’, not significant, Mann–Whitney test; *P<0.05, ‘n.s.’, not significant, ANOVA) (**d**) OT-I CTL were co-cultured with IFN*γ*R^+/+^ or IFN*γ*R^−/−^ (**e**) KC. Co-cultures were treated with anti-IFN*γ* or PBS. Included in each experiment were control wells: with isotype antibody (Iso), without peptide (No P), and without T cells (No T). (**f**) OT-I CTL were co-cultured with IFN*γ*R^+/+^ or IFN*γ*R^−/−^ (**g**) KC and were treated with rIFN*γ*, or rIFN*γ* and anti-IFN*γ*, or rIFN*γ* and isotype antibody. (**h**) IFN*γ*^−/−^ CD8 T cells were co-cultured with SIINFEKL-loaded B6 KC. Cultures were treated with rIFN*γ*, or rIFN*γ* and anti-IFN*γ*, or rIFN*γ* and isotype antibody

Death of KC targets *in vivo* is slow and not readily amenable to direct intravital imaging.^28, 29^ We have previously used prolonged time-lapse imaging of primary target and effector cells *in vitro* to show that the mechanism of killing was associated with T-cell kinematics.^28^ We utilized this method to determine the role of IFN*γ* in CTL cytotoxicity to keratinocytes. We isolated EGFP^+^CD8 T cells from mice primed to OVA *in vivo,* and from naive mice. As expected, IFN*γ* expression was high in primed cells from IFN*γ*-competent mice, when compared with naive cells, and undetectable in primed cells from IFN*γ*^−/−^ mice. However, CD44, a marker of T-cell activation, was elevated in primed cells from both IFN*γ*-competent and IFN*γ*^−/−^ mice (Figures 3a and b). Primed cells were co-cultured with primary keratinocytes loaded with OVA and imaged for 30 h (Supplementary Movie S3). OVA-primed CD8 T cells from both OT-1 and B6 mice killed KC with statistically similar efficiencies (Figure 3c). However, IFN*γ*^−/−^ CD8 T cells killed markedly less efficiently than IFN*γ*-competent cells (*P*<0.005 B6 *versus* IFN*γ*^−/−^; Figure 3c). The addition of anti-IFN*γ* to a co-culture of IFN*γ*-competent CTL and antigen-loaded KC resulted in markedly reduced target cell killing (Figure 3d). These data suggest that killing of KC by CTL was enhanced in the presence of IFN*γ*. If KC from IFN*γ*R^−/−^ mice were used as targets, killing by primed IFN*γ*-competent CTL was overall less efficient (Figure 3e), but was further inhibited by anti-IFN*γ* antibody (Figure 3e). This suggests that both IFN*γ*-mediated KC killing, and non IFN*γ*-mediated killing by CTL were affected by locally supplied IFN*γ*.

*In vivo*, IFN*γ* is supplied by many cells and is thus available via paracrine signaling to infiltrating CD8 T cells. To determine further the effect of locally available IFN*γ* on CTL killing, recombinant IFN*γ* (rIFN*γ*) was added to co-cultures of antigen-loaded KC targets and CTL. We first confirmed that at the concentrations of rIFN*γ* that we used (0.1 ng/ml), there was no increased death of KC above baseline in the absence of CTL in the co-culture (Supplementary Figure 1). Addition of rIFN*γ* to effector-target cultures with IFN*γ*-competent CTL resulted in a small but significant increase in cytolytic function – which was abrogated by the addition of anti-IFN*γ* (Figures 3f and g). Addition of rIFN*γ* to co-cultures of KC and IFN*γ*^−/−^ CTL also augmented killing of KC by IFN*γ*^−/−^ CTL (Figure 3h), suggesting that paracrine IFN*γ* affected activated CD8 T cells, independent of whether they produced the cytokine themselves. The levels of killing did not reach that of IFN*γ*-competent CTL (Figure 3c), however, suggesting that, although IFN*γ*^−/−^ T cells are competent at cytolysis when supplied with IFN*γ* (Figure 3h; *P*=0.032 rIFN*γ* *versus* PBS), the production and autocrine signaling of IFN*γ* may be important to T-cell cytolytic function.

### IFN*γ* directly promotes CTL cytotoxicity

**Figure 4:**
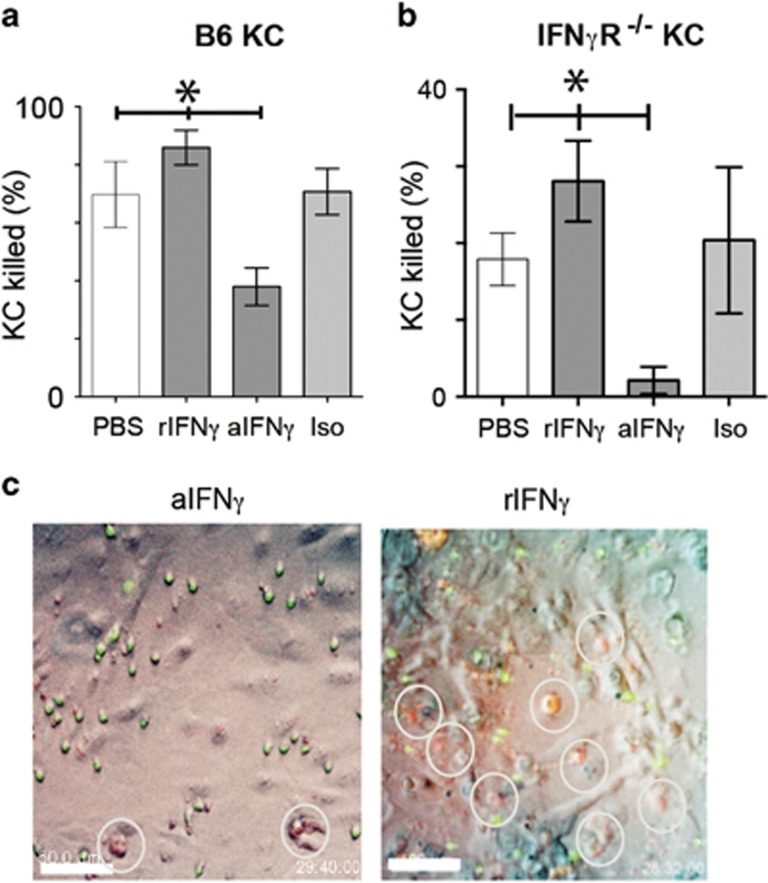
Modulation of IFN*γ* signaling in CTL affects cytotoxic function. EGFP^+^OT-1 CTL were incubated with PBS, rIFN*γ*, anti-IFN*γ* or isotype antibody for 4 h, then washed thoroughly and added to SIINFEKL-loaded (**a**) B6 or (**b**) IFN*γ*R^−/−^ KC along with indicator dye for activated caspase. Co-cultures proceeded for 30 h, and were imaged by fluorescence time-lapse microscopy. Keratinocyte death was determined as in Figure 3. (*P<0.05, ‘NS’, not significant, ANOVA). (**c**) Frames of representative fields of co-cultures after 28 h of imaging. Merged red, green and brightfield images shown. Dead KC fluoresce red. EGFP^+^ CTL are green. Dead KC are shown by white rings. Scale bar is 50 *μ*m

IFN*γ* up-regulates antigen presentation on epithelial cells. To determine whether in our model IFN*γ* was acting directly on CTL or on targets, we pre-treated CTL with IFN*γ*. OVA-primed CTL were treated for 4 h with rIFN*γ*, and then co-cultured with KC and imaged for 30 h. CTL pre-stimulated with rIFN*γ* killed targets significantly better than untreated T cells (Figures 4a and c) even when targets did not express the IFN*γ*-receptor (Figure 4b). CTL lysis of KC is thus enhanced by IFN*γ*, even when the KC cannot respond to secreted IFN*γ*. We then investigated whether IFN*γ* production by effector T cells enhanced their own effector function. We pre-incubated IFN*γ*-competent primed CTL with anti-IFN*γ* antibody, which is not cell permeable, for 4 h, washing cells thoroughly before co-culture with KC target cells. Killing of target KC was significantly inhibited, but not abrogated, by anti-IFN*γ* pre-treatment of CTL (Figures 4a and c). There was no recovery in levels of cytotoxicity over 30 h. We co-cultured antibody-treated CTL with IFN*γ*R^−/−^ KC to assess the effect of IFN*γ* inhibition on non-IFN*γ*-mediated cytotoxicity. In this case, cytotoxicity was almost completely abrogated, suggesting that CTL may be reliant on IFN*γ* at a metabolic level to effect any killing, even non-IFN*γ* cytokine mediated killing.

### IFN*γ* promotes CD8 T-cell motility

**Figure 5:**
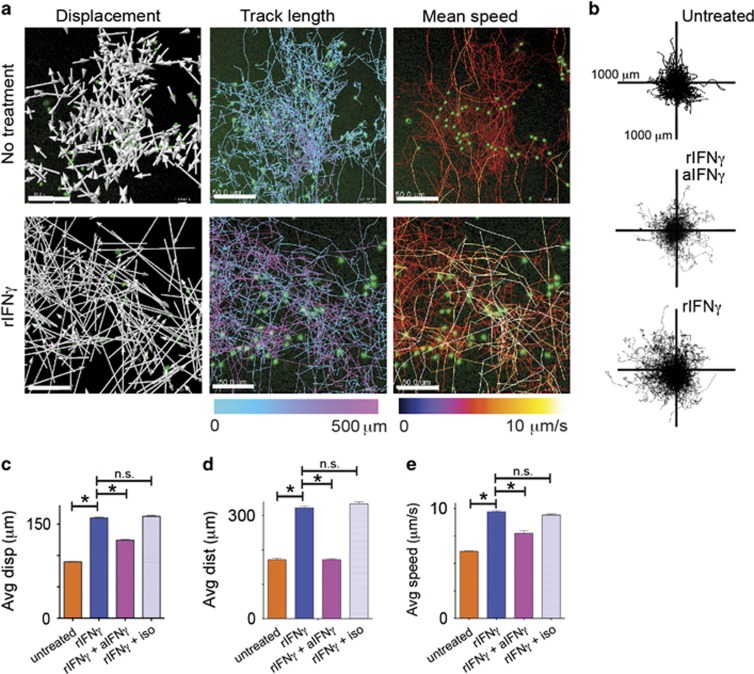
Exogenous IFN*γ* promotes CD8 T-cell motility and enhances cytotoxicity. (**a**) Co-cultures of EGFP^+^OT-I CTL and SIINFEKL-loaded or untreated KC were imaged for 30 h by fluorescence time-lapse microscopy. Recombinant IFN*γ* was added at time 0. Representative images of tracks created by CTL as they traveled among KC targets have been mapped by software. Vector displacement of the tracks is shown by white arrows. Length of the traveled tracks and mean speed of travel is indicated by the colored legend bars (scale bars, 50 *μ*m). (**b**) Translated travel tracks from (**a**) have been plotted from origin showing the distance and spread of the path traveled by the cells within the field of view. Each plot shows the result for one typical experiment of three independent experiments. (**c**–**e**) Displacement (**c**), distance traveled (**d**) and speed (**e**) of CD8 T cells in co-culture with antigen-loaded target KC. Average values of four movies are shown (>400 cells). Error bars are S.E.M. (*P<0.05, n.s. not significant)

Time-lapse movies of target-effector cell co-cultures were analyzed for CTL displacement, track length and mean track speed (Figure 5a and Supplementary Movie S2) The net patrol area of CTL increased with rIFN*γ* treatment, and was reduced with inhibition of IFN*γ* (Figure 5b). The addition of rIFN*γ* to the co-cultures markedly increased CTL vector displacement (rIFN*γ* mean 95 *μ*m; untreated mean 55 *μ*m; *P*=0.043, K–S test) and distance traveled (rIFN*γ* mean 254 *μ*m; untreated mean 218 *μ*m; *P*=0.023, K–S test; Figures 5c and d). CTL treated with IFN*γ* traveled with average speeds of 0.41 *μ*m/s, significantly faster than untreated cells (average 0.33 *μ*m/s; *P*=0.044, K–S test; Figure 5e). The addition of anti-IFN*γ*, but not isotype antibody, inhibited the stimulatory effects of rIFN*γ*.

**Figure 6:**
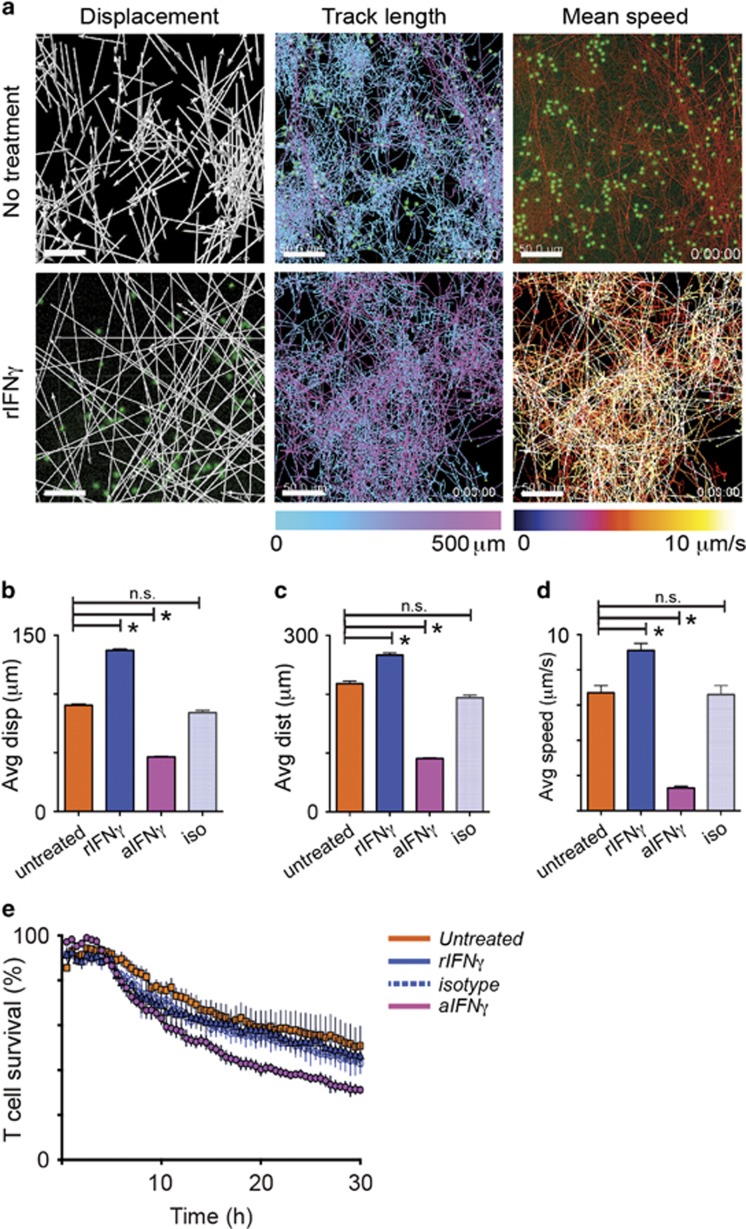
Suppression of autocrine signaling of IFN*γ* on CTL inhibits their cytotoxicity and motility. IFN*γ*^−/−^ CTL cells or EGFP^+^OT-I CTL pre-treated with PBS, rIFN*γ* or anti-IFN*γ* for 4 h, were co-cultured with SIINFEKL-loaded target KC. The kinematics of CTL were analyzed using fluorescence time-lapse movies of 30 h as above. (**a**) Examples of images of tracked CD8 T cells. Displacement is shown in white arrows, and track length and mean speed are shown in color. Scale bars, 50 *μ*m. (**b**–**d**) Pre-treated B6 CTL or IFN*γ*^−/−^ CTL in co-culture with targets for 30 h were investigated for vectorial displacement, distance traveled, and mean speed. Data are pooled results of 3 independent experiments (around 200 cells). Error bars are SEM. (*P<0.05, n.s. not significant) (**e**) Rate of death of pre-treated CD8 T cells during co-culture with target cells. The number of surviving T cells was determined by software, expressing the number of green cells (size >7 *μ*m) at each frame as a percentage of the number of cells at the start. *N*=3. Error bars are s.e.m.

To establish whether the increased motility was due to the effects of IFN*γ* on effector cells or on KC, CTL were pre-treated with anti-IFN*γ*, or with rIFN*γ* for four hours prior to co-culturing with targets, as above (Figure 6a). Treatment of T cells with rIFN*γ* markedly increased their vector displacement (mean 137 *μ*m, *P*<0.05 cf. untreated; Figure 6b) and distance traveled (mean 218 *μ*m, *P*<0.05 cf. untreated; Figure 6c). Likewise, cells moved faster with IFN*γ* pre-treatment (Figure 6d), with average speeds of 0.52 *μ*m s^−1^ (mean *P*<0.05 cf. untreated). IFN*γ* thus directly promotes CD8 T-cell motility. Pre-treatment of CD8 T cells with anti-IFN*γ* for four hours may be predicted to have little effect on CD8 T-cell kinematics as the cells would produce IFN*γ* in response to antigen presentation by KC. We found 4 h of pre-treatment with anti-IFN*γ* inhibited CTL motility: displacement, track length and mean speed were significantly reduced, while treatment with an isotype control antibody for the same amount of time had no effect. (Figures 6b–d).

We investigated whether IFN*γ* had an effect on T-cell survival, which in monoculture is >85% at 30 h (not shown). We showed previously that CTL loss accompanied target cell loss, usually after prolonged periods of attachment.^28^ Neither pre-incubation with rIFN*γ*, nor addition of rIFN*γ* to the co-culture, altered the rate of apoptosis of CTL. Pre-incubation with anti-IFN*γ*, however, resulted in a significant (*P*<0.05 cf. untreated and isotype) reduction of CD8 T-cell survival over 30 h (Figure 6f). CTL-derived IFN*γ* seems to contribute to T-cell survival of IFN*γ*-producing cells by autocrine signaling.

**Figure 7:**
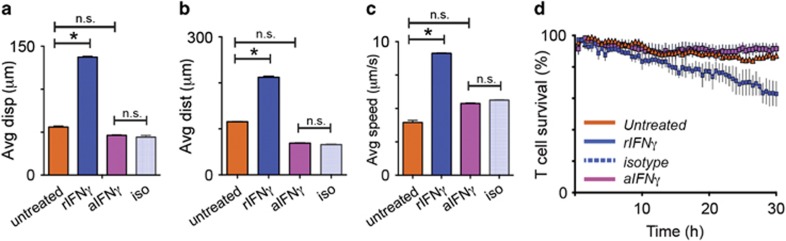
IFN*γ*-deficient cells show reduced kinematics but respond to IFN*γ*. CTL from OVA-immunized IFN*γ*-/- mice were pre-treated with rIFN*γ* or aIFN*γ* or isotype control antibody for four hours prior to co-culture with KC presenting SIINFEKL peptide. Cultures were imaged for 30 h as above. (**a-c**) Movies were analyzed for CTL displacement, track length, and average track speed. (**d**) CTL survival was calculated by comparing the number of CTL in the final imaging frames with those in the initial frames. (*P<0.05, n.s., not significant)

We next investigated these effects on IFN*γ*-deficient T cells. IFN*γ*^−/−^ *in vivo* OVA-primed T cells from IFN*γ*^−/−^ mice were pre-incubated with rIFN*γ*, or aIFN*γ* for 4 h prior to co-culturing with target KC expressing SIINFEKL peptide. Without treatment, IFN*γ*^−/−^ T cells displayed reduced vectorial displacement (89.8 *μ*m *versus* 56.0 *μ*m, *P*<0.05) and total travel distances (85.7 *μ*m *versus* 57.8 *μ*m, *P*<0.05) compared with IFN*γ*-competent CTL (Figure 7a and b). Their track speed was also slower (0.36 *versus* 0.64 *μ*m/s, *P*<0.05; Figure 7c). Incubating IFN*γ*-deficient cells with rIFN*γ* markedly enhanced CTL kinematics, bringing them up to levels comparable to IFN*γ*-competent cells similarly treated with rIFN*γ* indicating these cells responded similarly to locally supplied IFN*γ*. Incubation with aIFN*γ* had no effect on the behavior of IFN*γ*-deficient cells. While inhibiting IFNγ in IFN*γ*-sufficient cells reduced T-cell survival, untreated and antibody-treated IFN*γ*-deficient cells displayed minimal loss. Conversely, Treatment with rIFN increased CTL mortality, reflecting activation-induced cell death associated with increased killing ability (Figure 7d and 3h).

## Discussion

*In vivo* and *in vitro*, CTL kill target cells after a period of prolonged contact and at a low rate.^28^,^30^ Using a reductionist two-cell system, we now describe CTL-mediated killing of targets that is reliant on IFN*γ* at multiple interfaces including recruitment, motility, killing and survival.

IFN*γ*-deficient animals were unable to reject skin grafts expressing ovalbumin, despite a full complement of T cells (Figure 1b). Although some studies have suggested that in an IFN*γ*-deficient environment, priming is suboptimal,^31, 32, 33^ the presence of host cells in graft tissue after immunization suggested priming was occurring. It was more likely therefore that IFN*γ* was necessary for CTL cytotoxic function. CD8 T cells without IFN*γ*R2 can’t effect cytolysis in a chromium release assay, although they express Fas, perforin and IFN*γ*.^34^

IFN*γ* is a potent chemoattractant for CD8 T cells in inflamed tissue, amplifying antiviral responses.^35^ However, in skin, IFN*γ* has been reported as redundant for CTL recruitment or efficacy of function.^36^ We found transferred pre-primed IFN*γ*-competent CD8 T cells sufficient to reject fresh grafts and consistent with previous work showing the resistance of grafts to rejection without local inflammatory stimuli CTL also partially rejected well-healed grafts.^37^ Since inhibition of IFN*γ* increased CTL death *ex vivo*, it may also reflect a shorter lifespan of transferred cells in an IFN*γ*-deficient environment. We note that the lack of IFN*γ* expression in tissues resulted in fewer CD8 T cells migrating to the inflammatory site (Figure 1e), however they were sufficient to effect rejection. These effects may be due to *in vivo* reduction in antigen-expression by KC in an IFN*γ*-deficient environment, although the restoration of graft rejection by transfer of IFN*γ*-competent CTL suggests antigen presentation was sufficient.

IFN*γ* has many tissue-specific effects in inflammation including enhancing CD8 T-cell migration and priming in tissue by antigen-presenting cells, upregulating MHC-I expression on targets, all of which enhance CD8 T-cell-mediated target cell apoptosis in inflammation. However, rather than enhancing T-cell motility, inflammation appears to lead to arrest of CD8 T cells early in the rejecting skin graft, noted by us and others.^29^ Late in the process, when there is less tissue edema and inflammation, CTL are highly motile; less so in an IFN*γ*-deficient environment (Figure 3). We speculate that early in graft rejection, local factors such as hypoxia and edema may contribute to limiting motility of IFN*γ*-competent CTL, which are inherently highly motile, and whose kinematics are further enhanced by circulating IFN*γ*. Previous studies have shown the presence of antigen slows CTL, with increase in T-cell motility when antigen levels were reduced.^38^ Rejected graft sites no longer have antigen, and thus the activity of the local CTL in the region reflects their motility in the absence of antigen, while within intact grafts, antigen is slowing down the CTL. Nevertheless, IFN*γ*^−/−^ recipients do not reject OVA grafts, implying a deficiency in T-cell function, which may also be reflected in their motility.

The enhanced kinematics of CTL in response to IFN*γ* resulted in the cells covering a larger migratory region, both in vector distance, and by track length taken, both *in vivo* and *in vitro* (Figures 2 and 5). These aspects of enhanced motility suggest an increased likelihood of target cell being encountered. It was speed which most closely correlated with function, particularly illustrated by treatment with anti-IFN*γ* (Figure 5e). It is not clear whether inhibition of autocrine IFN*γ* lead to metabolic disturbances resulting in both cell slowing and impairment of killing, or whether the slowing down of the cells merely inhibited their ability to seek and kill targets. As T cells were incubated with a large excess of targets in our co-culture systems, we speculate the former is more likely.

Although KC are sensitive to non-contact dependent IFN*γ*-mediated killing, the production of IFN*γ* by effector cells did not appear to be sufficient to kill bystander cells here, as evidenced by lack of KC death in co-cultures without peptide-loading (Figures 3d and e). Secretion of IFN*γ* by antigen-specific CD8 T cells in a similar co-culture system demonstrated activation of IFN*γ*-dependent STAT1 in bystander cells but formation of an immunological synapse was crucial to the release of IFN*γ*, indicating antigen-specificity of IFN*γ* release.^39^ This finding is consistent with our data that activated CD8 T cells do not kill KC unless they express cognate antigen, and that KC deficient in IFN*γ* receptor are more resistant to cell-mediated cytolysis. We observed almost no apoptosis of KC, as evidenced by caspase activation, without direct contact of KC with effector cells, although there may have been non-cytolytic STAT1 activation in surrounding cells not detectable in our assay. Additionally, IFN*γ* has been shown to stimulate production of pro-inflammatory cytokines such IL-33 – a member of the IL-1 family – in keratinocytes.^40^ IL-1 directly enhances the activation of antigen-specific OT-1 CD8 T cells *in vivo* as evidenced by migration into peripheral tissues and *in vivo* cytotoxicity assays.^41^ This suggests that target keratinocytes may have the ability to directly enhance CD8 T-cell cytotoxicity in an inflammatory environment, and may be the mechanism by which CD8 T-cell kinematics and cytotoxic function are enhanced in our model.

Pre-incubation of CD8 T cells with IFN*γ* directly enhanced CD8 T-cell cytolysis independent of its effects on target cells. CD8 T cells not expressing the IFN*γ*R are not able to lyse antigen-expressing cells, indicating the cytolytic function of these cells is dependent upon their IFN*γ*R.^34^ By providing an environment rich in IFN*γ*, CD4 T helper 1 cells and natural killer T cells can also potentially enhance CTL cytolytic function in inflammation. By corollary, production of downregulators of IFN*γ* may directly reduce CD8 cytolysis, a pathway by which tissues may protect themselves from inflammatory damage.^42^ IFN*γ* stimulates the production of other cytokines from cells in an inflamed environment including IL-1 by innate immunocytes, which has been shown to markedly enhance the antiviral function of CD8 T cells.^41^ We speculate that this may be another mechanism by which IFN*γ* released into the local environment works to enhance function of CTL themselves. CD8 effector cells in tissue vary in their sensitivity to IFN*γ*, likely reflecting different populations, not all of which are equally cytolytic,^43^ suggesting a potential target for immune modulation.

IFN*γ* has been purported to play a key role in antigen-induced T-cell death.^44^ The modest but significant increase in motility kinetics and cytolytic activity of CD8 T cells post IFN*γ* treatment likely reflects intracellular metabolic changes induced by the cytokine. Prolonged IFN*γ*-deprivation, achieved by a depleting antibody, resulted in irreversible changes to CD8 T-cell function, suggesting that CD8 T cells heavily rely on autocrine IFN*γ* to maintain cytolytic and kinetic function. Granzyme B and IFN*γ* expression was impaired in the absence of IRF3 in murine models.^45^ Activation of the TCR together with its co-receptor has been shown to result in activation of STAT proteins within the cell one hour later, suggesting that any metabolic effects of IFN*γ* are likely to be slow rather than immediate,^46^ in keeping with our data where cells were treated for 4 h with inhibitor.

We describe a process of CD8 T-cell-mediated antigen-specific killing of keratinocytes that is reliant on IFN*γ* at multiple interfaces. The cell-specific resistance to CTL exhibited by keratinocytes is related to their susceptibility to IFN*γ*, and mediated by the IFN*γ*R. We also show that CD8 T cells are themselves up regulated by IFN*γ*, and that these effector cells are reliant on their ability to produce IFN*γ* for their own function and survival. Further studies *in vivo* examining these mechanisms utilizing systemic blockade of IFN*γ* and local and systemic supply of the cytokine will be of value. The reliance of the CTL-mediated killing of keratinocytes on IFN*γ* at multiple levels emphasizes its importance as a mediator of effector T-cell function.

## Materials and methods

### Mice

Experiments were approved by the University of Queensland animal ethics committee. B6.K5mOva (K5mOva) mice were a kind gift from Dr W.R. Heath, WEHI, Parkville, Australia. B6.OT-I and B6.RAG-1^−/−^ mice were purchased from the Animal Resource Centre, Perth, Australia. B6.IFN*γ*^−/−^ and B6.IFN*γ*R^−/−^ mice were purchased from Jackson Laboratories, Bar Harbor, ME, USA. B6.Nzeg (EGFP^+^B6) mice were bred with OT-I mice to create OT-1.Nzeg (EGFP^+^OT-I) mice, and with B6.IFN*γ*^−/−^ to create IFN*γ*^−/−^.Nzeg (EGFP^+^IFN*γ*^−/−^) mice. We confirmed the EGFP expression did not act as an alloantigen by the acceptance of skin grafts from these mice onto C57/B6 (B6) mice, and by co-culture of EGFP^+^ lymphocytes with B6 lymphocytes which did not result in increased cell death.

### Reagents

The following antibodies were purchased from BD Pharmingen (San Jose, CA, USA): anti-CD44 APC-Cy7, anti-IFN*γ* PE-Cy7, CD8 PE, and CD8 APC, relevant isotype antibodies. Anti-CD62L PE and anti-IFNγ capture antibody (Clone R4-6A2) were purchased from eBioscience (San Diego, CA, USA). Anti-GFP FITC was purchased from Abcam (Cambridge, UK). SR-FLIVO-red was courtesy of ImmunoChemistry laboratories (Bloomington, MN, USA). All cell culture media was purchased from Invitrogen (Carlsbad, CA, USA). Recombinant IFN*γ* was purchased from R&D Systems (Minneapolis, MN, USA). Z-DVED-FMK, Ovalbumin (OVA) and SIINFEKL peptide were purchased from Sigma-Aldrich (St. Louis, MO, USA).

### Mouse experiments

For grafting, mice were used between 6 and 8 weeks of age. Adoptive transfer of cells was performed by tail vein injection of 10^6^ cells concentrated into 100 *μ*l of saline. Administration of antibody was performed by intraperitoneal injection of 200 *μ*g anti-IFN*γ* or isotype control antibody, diluted into 200 *μ*l saline administered at the time of adoptive transfer and weekly thereafter.

### Skin grafting

Grafting of murine ear skin to flank was performed as previously described.^47^ Briefly, donor ears were split antero-posterior and the cartilage scraped. A comparable size of skin was excised from the recipient mouse and the graft was placed dermis-side down over the deficit. The graft was bandaged in place for 7 days. Grafts were deemed rejected if there was >80% loss of epithelium, and as accepted if there was no rejection by day 50.

### Activation of CD8 T cells

Mice were immunized with OVA (50 *μ*g) and QuilA (20 *μ*g), or mock-immunized, subcutaneously at the base of the tail. 10 days later, isolated CD8 T cells from immunized mice were cultured for 3 days in complete RPMI, with IL-2 10 ng/ml, and SIINFEKL peptide 1 *μ*g/ml. Activation of cells was confirmed by flow cytometry for CD8^+^CD44^high^ T cells. In adoptive transfer experiments, 10^6^ CD8^+^CD44^high^ T cells were injected by tail vein.

### Keratinocyte harvest and culture

Primary KC were harvested from neonatal mice aged 3–4 days as described previously.^48^ Briefly, skins were removed from sacrificed mouse pups and digested overnight at 4 °C in 10% dispase solution containing 1% penicillin, streptomycin and gentamycin, before brief trypsinisation and resuspension of separated primary KC in DMEM. KC were seeded at 30–50% confluence in DMEM with 5% FCS for 24 h, onto 24- or 47-well tissue culture plates. Media was changed to keratinocyte serum-free medium supplemented with bovine pituitary extract and epidermal growth factor 24 h later. Cells were used for imaging at 60–70% confluence.

### Co-culture experiments

SIINFEKL peptide 1 *μ*g/ml was added to the KC medium for 1 h at 37 °C, and cells then washed 3 times in PBS to remove non-incorporated peptide. CD8 T cells 5 × 10^3^ were added per well in duplicate wells. Cells were imaged at 37 °C with 5% CO_2_ for 30 h in imaging media, comprising 45% RPMI, 5% FCS, 50% serum-free medium, 5 ng/ml IL-2 and 2% FLIVO-SR red solution. Each plate included control wells of co-cultures without peptide and KC monocultures. Where indicated, recombinant IFN*γ* (1 *μ*g/ml), anti-IFN*γ* (1 *μ*g/ml), or isotype control antibody were added to the media.

### Flow cytometry

Single-cell suspensions of leukocytes were prepared with complete RPMI inclusive of 5% FCS, 1% penicillin and streptomycin. Pelleted cells were lysed with ACK lysis buffer (Invitrogen), and washed in sterile FACS-buffer comprising PBS with 5% FCS. For T-cell selection, resuspended cells were stained with anti-CD8 APC antibody, and 1 *μ*g propidium iodide (Invitrogen). FACS was used to obtain a >98% pure population of APC^+^, PI^-^ cells (Moflo, Beckman Coulter, Brea, CA, USA). Cells were washed in sterile PBS in preparation for transfer to recipient mice by tail vein injection, or resuspended in complete RPMI for culture. For flow cytometric examination, surface staining of single-cell suspensions were performed at 4 °C before fixation in 1% formalin in PBS. Where intracellular cytokine staining was required, T cells were pre-stimulated with 25 ng/ml PMA/ionomycin (Sigma-Aldrich) in the presence of 1 *μ*g monensin (Sigma-Aldrich) for 4 h at 37 °C. Cells were then put on ice and surface stained, before incubation in fixation and permeabilisation buffer (BD Systems, Franklin Lakes, NJ, USA) for 30 min before intracellular staining. Flow cytometry was performed using FacsCanto (BD Systems).

### Immunofluorescence

Grafted mice were injected intravenously with FLIVO-SR red prior to sacrifice. Frozen sections of excised skin grafts of mice receptive of adoptively transferred CD8 T cells were stained with GFP-conjugated anti-CD8 antibody 2 h at 4 °C before fixation in 4% paraformaldehyde (ProSciTech), mounting and examination. In experiments where intravenous FLIVO-SR red was not used, these slides were stained with Alexa 555-conjugated anti-CD8 antibody and FITC-conjugated anti-GFP antibody, before fixation and mounting.

### Microscopy

Confocal microscopy of slides was performed on a Zeiss (Oberkochen, Germany) Meta-250 confocal microscope with × 25 oil objective lens, taking 2 *μ*m Z-sections. Extended-duration time-lapse microscopy was performed on a Zeiss Axiovert 200M microscope acquiring images using a Zeiss AxioCamMR camera. Images binned 4 × 4 were acquired from duplicate wells using a × 10 objective lens. Cells were maintained with humidification, in 5% CO_2_, at 37 °C during imaging. Images were taken every 12 min for 30 h. We have previously optimized the imaging frequency of these co-cultures to obtain maximal duration of imaging with no detectable phototoxicity.^28^ While the track length and mean track speed depend on imaging frequency, as the same time gaps between images were used for all cultures, including controls, and as each imaged culture plate comprised all controls and experimental samples, the variation is assumed to be similar in both treated and control wells. Indeed in earlier studies we confirmed that imaging with increasing frequency made little difference to mean track length or speed over 4 h of imaging, but markedly increased culture cell death due to phototoxicity seen as cell membrane damage and porosity.^28^

### Multiphoton microscopy

Mice anaesthetized with inhaled isoflurane, were warmed on a heating pad regulated by core rectal temperature measurements. A longitudinal incision was made either side of the graft and a glass slide was used to elevate the flank skin. A coverslip in a heated chamber was placed over the graft. A LaVision multiphoton microscopy system with Mai Tai laser with a × 16 dipping objective lens was used to acquire images. Images were acquired every 60 s for 15–30 min. Where a graft was still in place, imaging was performed at the edge of the graft; where a graft was rejected, the site of graft placement was imaged.

### Image analysis

Imaris 7.4.2 software (Bitplane, Ulster, UK) was used to de-convolute and analyze images, and generate movies. Numerical data were analyzed using raw images. Analysis parameters were optimized for each image, to minimize mis-tracking. T cells were identified using the spot selection and tracking software by size (7 *μ*m) and EGFP expression, and tracked through each frame. We excluded tracks within 10 *μ*m from the edge, and tracked with maximum gap size 1, and distance 20–50 *μ*m. Spots and tracks were manually corrected in up to 10% of cells per field. Cell death was determined by fluorescence in the red channel and size: KC 20 *μ*m, CTL 7 *μ*m. Data were exported into Excel and into Graphpad Prism for statistical analysis. Spectral separation was where required, and a 3 × 3 median filter and minor contrast adjustments were performed using Imaris or Adobe Photoshop on still images and movies for publication. Further details regarding parameters used for analysis, spot selection, Imaris algorithms and validation of the techniques have been previously described in detail.^28^

### Statistics

Intravital imaging data were collected from multiple sites per animal and averaged. Cell culture data were collected from duplicate wells and averaged. *N* refers to the number of independent experiments performed. We used the one-tailed Mann–Whitney test to compare non-parametric data, the Kolmogorov–Smirnov comparative test (K–S test) to compare two frequency distributions that were non-normal and the Mantel–Cox test was applied to survival data as indicated in the text. Data were considered significantly different if *P*<0.05.
