# Supplementary figures and images for: Interferon-γ derived from cytotoxic lymphocytes directly enhances their motility and cytotoxicity
Source: Cell Death Dis. 2017 Jun 1;8(6):e2836–. doi: 10.1038/cddis.2017.67 (PMC5520949; doi:10.1038/cddis.2017.67)

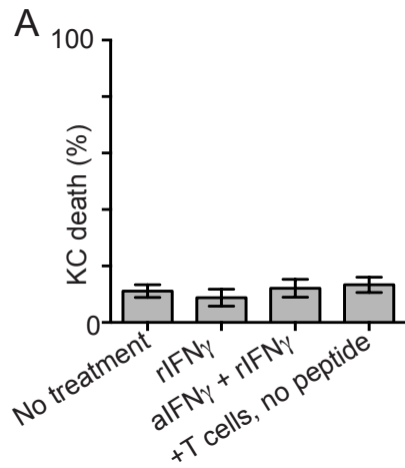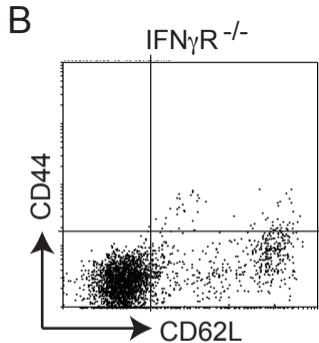

Supplement: Supplementary Figure S1 [file cddis201767x1.pdf]
