# Supplementary material for: Interferon-γ derived from cytotoxic lymphocytes directly enhances their motility and cytotoxicity
Source: Cell Death Dis. 2017 Jun 1;8(6):e2836–. doi: 10.1038/cddis.2017.67 (PMC5520949; doi:10.1038/cddis.2017.67)
Supplement: Supplementary Legends [file cddis201767x5.docx]

SUPPLEMENTARY FIGURE LEGENDS

**Supplementary Figure 1. Controls. A.** CTL were co-cultured with target KC and the effect of IFNγ was determined by either pre-treatment of the effectors, or by adding to the co-culture. Here we show control wells confirming specificity of the result. Addition of 0.1 μm of IFNγ to KC in monoculture did not cause increase in death of the KC in the absence of T cells. Likewise, the addition of both antibody to IFNγ and rIFNγ did not increase KC death in the absence of T cells. In the presence of activated T cells, no KC killing was observed in the absence of presentation of cognate peptide. **B.** IFNγR^-/-^ mice are unable to produce CTL after immunization intradermally with OVA. Immunisation of IFNγR^-/-^ mice by intradermal injection of OVA and QuilA adjuvant to tail base was performed 14 days prior to harvest of splenocytes. Cells were stained for activation markers and analysed by flow cytometry. Live lymphocyte-sized cells have been gated for CD8 expression.

**Movie S1:** **Example of** **intravital** **2-photon microscopy of CD8 T cells during skin graft rejection in B6 mice.** OVA skin grafts were placed on B6 mice adoptively transferred with EGFP^+^OT-1 CTL. At day 19, the grafts were almost completely rejected by inspection of the skin. Mice were anaesthetized and the periphery of the skin grafts was imaged by 2-photon microscopy. There is a significant number of EGFP^+^CTL that are seen moving rapidly among the tissue. **Tracked CTL during skin graft rejection in B6 mice.** Cells in the left panel were identified by software and tracked. Tracks are colour-coded to reflect speed, ranging from 0.002 – 10 μm.s^-1^.

**Movie S2:**  **Example of intravital** **2-photon microscopy of CD8 T cells during skin graft rejection in IFNγ^-/-^ mice.** OVA skin grafts do not reject when placed on IFNγ^-/-^ mice. IFNγ^-/-^ mice were adoptively transferred with EGFP^+^OT-1 CTL before grafting. At 19 days, there was almost complete graft rejection, as with B6 mice in Movie 1. Mice were anaesthetized and the edges of the skin grafts were imaged by 2-photon microscopy. A similar infiltrate of CD8 T cells is seen here as in the B6 recipients, also moving among the tissue. **Tracked CTL during skin graft rejection** **in IFNγ^-/-^ mice.** Cells in the left panel were identified by software and tracked. Tracks are colour-coded to reflect speed, ranging from 0.002 – 10 μm.s^-1^. CTL moved at a markedly slower speed in an IFNγ-deficient environment.

**Movie S3: Example of time-lapse fluorescence imaging of primary co-cultures of antigen-specific CTL and peptide-expressing KC targets.** EGFP^+^CTL were co-cultured for 30 hours with SIINFEKL-loaded KC targets. Cell-permeant dye FLIVO-red has been added to the media, which fluoresces a brighter red upon intracellular activation of caspases. Images were taken sequentially in brightfield, red and green channels and merged to present this movie. Small bright green CTL are visible among the larger, non-coloured and less distinct KC targets. Small red apoptotic T cells can be seen in the image. The larger red cells are apoptotic KC. **CTL motility in co-culture with peptide-loaded target cells.** The green channel in left panel is shown. EGFP^+^ cells 7 μm in size have been identified by the analysis software and tracked over 30 hours of imaging. Manual track correction was applied where required. Where there was some doubt as to the correct tract, the default selection was allowed. Tracks are colour-coded for mean track speed, as indicated in the legend in Figure 6.
